# Influences on the access to and use of formal community care by people with dementia and their informal caregivers: a scoping review

**DOI:** 10.1186/s12913-018-3825-z

**Published:** 2019-02-01

**Authors:** Anja Bieber, Natalie Nguyen, Gabriele Meyer, Astrid Stephan

**Affiliations:** Institute of Health and Nursing Sciences, Martin Luther University Halle-Wittenberg, Halle (Saale), Germany

**Keywords:** Access, Utilisation, Dementia, Community care, Influencing aspects

## Abstract

**Background:**

The literature describes the obstacles to sufficient care faced by people with dementia and their informal caregivers. Although factors influencing access and utilisation are frequently studied, the body of knowledge lacks an overview of aspects related to influence. The frequently used Behavioural Model of Health Care Use (BM) could be used to structure and explain these aspects. An adaptation of the BM emphasises psychosocial influences and appears to enrich the understanding of the use of long-term care for dementia.

**Methods:**

We conducted a scoping review with the aim of providing an overview of the aspects influencing the access to and utilisation of formal community care in dementia. Our search covered the PubMed, CINAHL, Social Science Citation Index and PsychInfo databases, as well as grey literature. Two researchers assessed the full texts for eligibility. A data extraction form was developed and tested. We analysed the main topics investigated by the studies and mapped and described the investigated psychosocial aspects according to the BM after narratively summarising the findings. We used the Mixed Method Appraisal Tool (MMAT) to critically appraise the included studies.

**Results:**

A total of 94 studies were included: *n* = 55 with quantitative designs, 35 with qualitative designs and four with mixed methods. The studies investigated different services, mainly focusing on health care services. One third of the studies provided information regarding the severity of dementia. The most frequently investigated main topics were ethnicity and attitudes towards services. Psychosocial aspects were frequently investigated, although few studies considered the perspectives of people with dementia. Approximately half of the studies reported a theoretical framework. The adapted BM facilitated the structuring and description of psychosocial aspects. However, this instrument did not address topics beyond the scope of psychosocial aspects, such as sociodemographic characteristics.

**Conclusions:**

The access to and utilisation of formal community care for dementia can only be partly explained by individual influencing aspects. Therefore, a theoretical framework would likely help to describe this complex subject. Our findings indicate that the psychosocial categories of the adapted BM enriched the original BM, and that people with dementia should more often be included in healthcare service research to ensure a better understanding of the barriers to accessing formal community care.

## Background

People with dementia face permanent decreases in their abilities to handle everyday tasks throughout the course of their disease. The effects on the families have been well described [1]. In the majority of cases, relatives of people with dementia take on the responsibility for providing care, which often turns out to be more stressful than caring for someone without cognitive deficits [2]. Although more intense care must be provided to people with dementia, compared to their counterparts without dementia [3], many informal caregivers are hesitant about using professional support [4]. Empirical findings suggest that people with dementia have comparably restricted access to care [5], which is attributed to both individual and systemic reasons. For instance, informal caregivers might not consider the need for services, and care recipients might be reluctant to use services or lack knowledge about available services [6]. People with dementia and informal caregivers often feel that the timeline for receiving professional support is unnecessarily prolonged [7]. At the systemic level, a lack of provision of services has been identified, as home care services are mostly not tailored to dementia [8].

Reviews related to the use of professional support by people with dementia often focus on specific aspects. A review of service use in rural and remote settings [9] found evidence for low service use, gaps in service provision or services which not always were appropriate for dementia specific needs. Increasing public awareness was recommended to overcome these barriers. Reviews focusing on the use of respite care [10, 11] identified barriers even when carers were informed about the services. Reasons were a lack of awareness of the need to have a break from caregiving responsibilities and available social support resources. Specific groups of users and non-users such as older adults from ethnic minority groups [12, 13], male caregivers [4, 14] or people with dementia and comorbidity [5] were targeted in further reviews. Very important challenges for services use by the specific minority group of South Asians in the UK were among others a limited understanding of dementia, and a lack of information about available services [12]. People with dementia from ethnic minority groups could have enhanced access to support services, if they would recognize dementia as an illness and have knowledge about it [13]. Male informal caregivers of people with dementia were hindered from service use by service-related aspects, and attitudes for seeking help [4]. Some male informal caregivers experienced their interactions with service providers as being not very helpful [14]. People with dementia and other chronic conditions might have even more pronounced barriers to treatment [5]. Models of care were often designed specifically for a specific chronic condition, not taking the needs of people with comorbidity into consideration.

Although quite a few reviews give insight into special aspects of access to and use of formal community care, a broad overview of the influencing factors is currently lacking. A literature review was conducted as part of the recent Actifcare study [16] to find evidence on socioeconomic and cultural influences on access to and utilisation of formal care in eight participating countries [15]. The findings suggested that among others psychosocial factors significantly influence access to formal care [17–19]. The small number of identified studies highlighted the need for a more comprehensive overview to reflect the research activities on this topic.

## Objective

This scoping review aims to provide an overview of previously investigated aspects influencing the access to and utilisation of formal community care by people with dementia and their informal caregivers. Furthermore, we aim to improve the understanding of the appropriateness of the adapted BM for describing the utilisation of professional support for dementia.

## Methods

### Theoretical background

Several theoretical models or concepts have been used to analyse the dimensions of informal and formal care for dementia [20]. One of the most frequently used models, the Behavioural Model of Health Service Use (BM) [21], has been revisited several times [22] and applied to dementia care [23, 24]. The first versions of the BM addressed individual characteristics of the health service user (i.e., predisposing factors), conditions that make healthcare resources available (i.e., enabling factors) and conditions of perceived and evaluated health (i.e., need factors) [25]. Although aspects related to service provision and informal caregivers have complemented the updated versions [26, 27], other aspects of healthcare utilisation remain unexplained [28], leading to suggestions that adding psychosocial aspects (e.g., belief factors) might improve the BM [10]. The original model considered individual health beliefs to be predisposing factors. However, the Theory of Planned Behaviour (TPB) [29] provides a more detailed explanation of belief aspects. This theory considers several beliefs as the prevailing determinants of a person’s intentions and actions. These beliefs are distinguished as behavioural (i.e., beliefs about the outcomes associated with service use), normative (i.e., the views of others regarding service use behaviours) and control beliefs (i.e., factors perceived as facilitating or hindering service use) [10]. All three types of belief are included in a modified version of the BM developed for long-term care settings [30]. In the adapted BM, the psychosocial aspects are categorised as attitudes, knowledge aspects, social norms and perceived control. Attitudes describe personal views concerning the use of long-term care services, and an interrelationship between knowledge and attitudes was identified as apparent and difficult to disentangle [30]. Regarding the knowledge aspect, Ajzen [29] argues that professionals should explore the information actually possessed by individuals and its effects on their intentions and actions. The adapted BM describes normative beliefs as social norms, particularly the perceived behavioural expectations of important resources (e.g., spouses or doctors). Control beliefs are mentioned in the domain of perceived control, which refers to individuals’ perceived abilities to influence their choices regarding long-term care. A description of the categories of the adapted BM is provided in Appendix. The research team described these factors based on the methodological literature of the BM [25] and the Theory of Planned Behaviour [31]. Bradley et al. [30] used the adapted version of the BM to explore the determinants of intended future service use. However, it remains unknown whether the adapted BM is useful for describing the access to and utilisation of formal community care in the context of dementia.

### Design

The scoping review was guided by the methodological framework devised by Arksey and O’Melley [32] and the recommendations of Levac et al. [33]. This methodology allows the inclusion of all types of studies and provides an overview of the breadth, rather than depth, of evidence [34, 35]. A scoping review does not require a quality appraisal of the included studies, and this step is generally not performed [32, 36]. However, whether the methodological quality of the included studies should be appraised remains controversial [33, 37], and approximately a quarter of scoping reviews published in 2014 included this step [38]. We therefore decided to include a critical appraisal and used the Mixed Method Appraisal Tool (MMAT) [39], a reliable and practical instrument [40, 41] designed for the appraisal stage of complex systematic literature reviews covering qualitative, quantitative and mixed method studies. The MMAT has been used in previous scoping reviews [42–44].

### Search methods

We searched the PubMed, CINAHL, Social Science Citation Index and PsychInfo databases for quantitative, qualitative, and mixed-method studies. Additionally, we screened documents from the World Health Organisation, Organisation of Economic, Co-Operation and Development and Alzheimer’s Disease International. The literature search was conducted in January 2016 and updated in November 2016. The following search terms were used: dement*, Alzheimer, professional care, care giving, home care, community care, care, formal care, long-term care, informal care, long-term support, formal support, utilisation, utilization, access, service use, service non-use, help-seeking and help seeking.

### Inclusion criteria

We included literature reporting the investigation of aspects influencing the access to and the utilisation of professional formal community care by people with dementia and their informal caregivers in private home settings. Formal care was defined as community care, and included health and social care services such as home care services, day care, counselling and respite care provided by a formally paid professional. English language publications published between 1995 and 2016 were included. Studies related to institutional, palliative, or medical care and those conducted from the perspective of health care professionals were excluded.

### Study selection

Initially, the titles and abstracts were screened by two independent researchers (AS and AB). Next, the researchers independently reviewed the full texts of positively screened studies to determine eligibility. Any disagreements regarding inclusion were resolved by discussion, and a third researcher was included if necessary. Reviews were read completely, and the included references were also assessed.

### Analysis

A data extraction plan was developed and subjected to a pilot analysis of completeness and applicability by two researchers. The following information was extracted from the literature: study design, study location, study population, dementia severity among study participants, theoretical framework and formal care service structure. We extracted data regarding the frequently investigated formal care structures of counselling, day care, education, home care, home help, respite care and other services. Counselling services were defined as the provision of professional advice for people with dementia and/or informal caregivers. Day care services were defined as the provision of care for home-dwelling people with dementia, either at home or in day care facilities. Education was defined as the attempt to improve knowledge about or develop skills for living with dementia or caring for someone with dementia. Home care services provided help with basic activities of daily living, such as bathing, dressing, eating and mobility. Home help services provided help with instrumental activities of daily living, such as housework, shopping, or cooking. Respite care was defined as care in a nursing home, residential living facility, or comparable setting to provide around-the-clock relief for an informal caregiver of a family member with dementia.

All of the studies were analysed with respect to the investigated influencing aspects, using a qualitative content analysis approach. This includes reduction, explication, and structuring of the material [45]. We reduced the material of the study reports to information regarding influencing factors. We explicated the context material to achieve a deeper understanding of the background aspects. The identified influences were thematically classified according to the investigated main topics, presented in a table (Table 3), and narratively described. The next step of the analysis was theory-oriented according to the categories of the adapted BM [30]. We aimed to analyse these influencing aspects, which are part of the model, and to identify the limitations of the model.

## Results

**Fig. 1:**
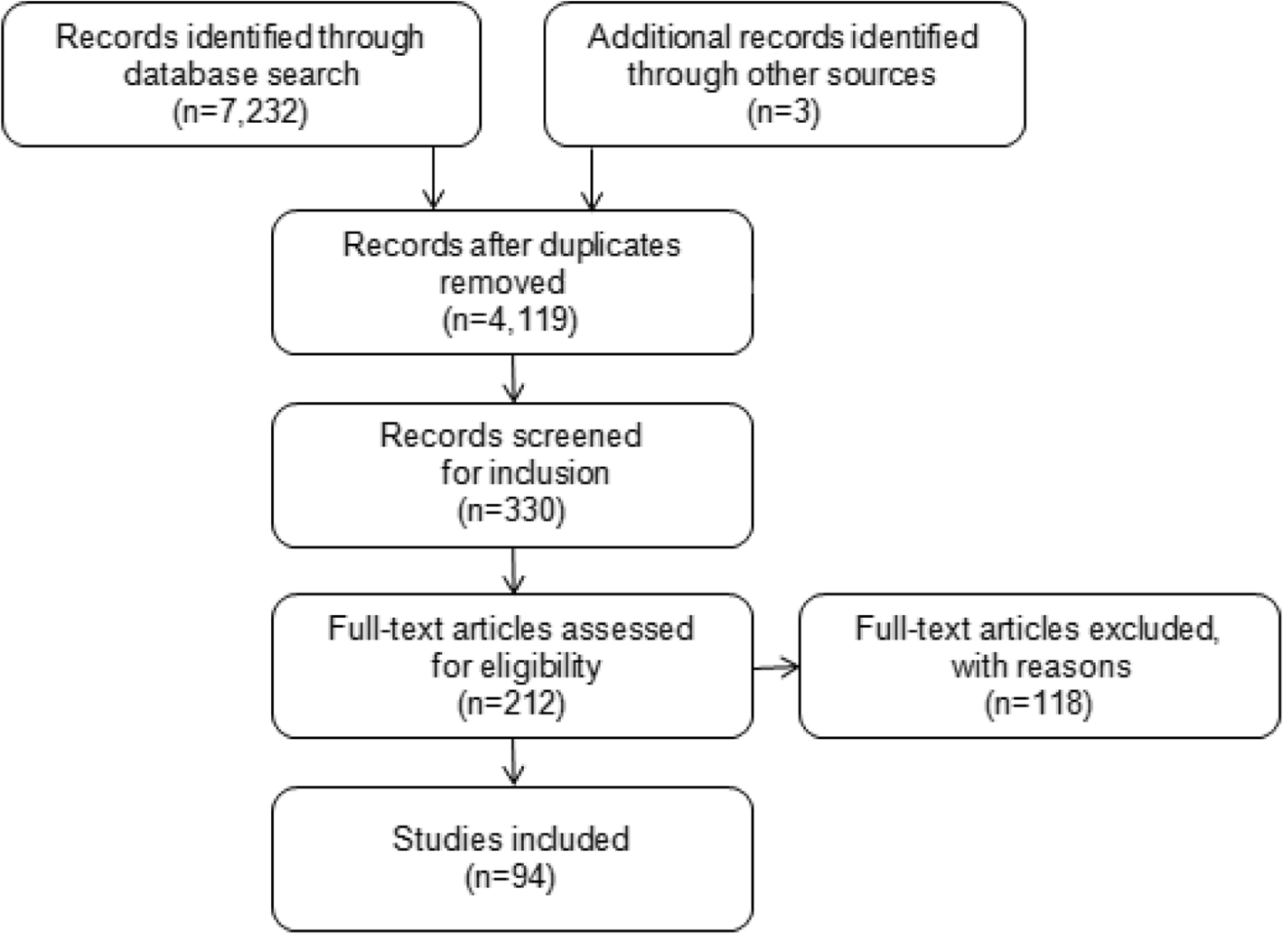
presents a flowchart of the study selection process

We included 94 references. An overview of the selection process can be seen in Fig. 1.

### Overview of study characteristics

#### Study design

More than half of the studies (*n* = 55) used a quantitative design. Nearly half of the studies (*n* = 45) collected cross-sectional data [17, 23, 27, 28, 46–88]. Six studies used a longitudinal design [87–92]. Four experimental designs were included (one randomised controlled trial [93] and three quasi-experimental studies) [94–96]. A third of the studies (*n* = 35) were qualitative and used one or more data collection methods. Twenty-eight studies included individual interviews [97–121]. Ten studies used focus groups [99, 115, 117–124], two comprised case studies [125, 126] and one analysed qualitative data from a questionnaire [127]. Four studies used mixed methods which combined quantitative and qualitative interviews.

#### Study location

The majority of studies were conducted in North America (*n* = 46), followed by Europe (*n* = 31), Australia (*n* = 13) and Asia (*n* = 4).

#### Study population

In two thirds of the sample [*n* = 61], the studies addressed the informal caregivers’ perspectives regarding the utilisation of services, and the caregivers therefore served as informants for the study. Of these studies, 29 had a quantitative design [17, 23, 28, 46, 49, 50, 54, 57, 59–62, 64, 66–75, 77–80, 88, 127], 29 had a qualitative design [82, 97, 100–108, 110–124, 128–130] and 3 had a mixed method design [131–133]. In one study addressing informal caregivers [81], patients with dementia were considered the informants for cognitive, functional and behavioural measures.

Twelve studies addressed both people with dementia and informal caregivers. Of these, eight were quantitative studies [47, 58, 86, 87, 90, 93–95] that included people with dementia as informants in addition to the informal caregiver’s perspective; however, the people with dementia provided information only about cognitive, functional and/or behavioural measures, and not service use. Only one quantitative study [85] included the perspectives of people with dementia regarding need aspects and service availability. Three qualitative studies [99, 109, 125] included people with dementia and informal caregivers, although Bowes [125] only included one person with dementia. In one mixed-method [134] and six quantitative studies [27, 63, 65, 83, 91, 135], only informal caregivers served as informants. One quantitative study [96] did not mention whether people with dementia were included in the service-related assessments.

Only 12 studies solely addressed people with dementia. Ten of these studies were quantitative; in half of this subgroup, people with dementia acted as informants only for cognitive measures or functional status [48, 52, 76, 84, 89]. Informal caregivers gave information on the influencing aspects in these studies. By contrast, the other five studies [51, 53, 55, 56, 92] asked people with dementia about their reasons for using services to some extent only. In the two qualitative studies [98, 126], people with dementia were the only informants.

#### Severity of dementia

One third of the studies presented information regarding the severity of dementia [47–49, 51–53, 58, 74, 76–80, 83–90, 92, 94–96, 98, 106, 109, 116, 129, 132, 134, 135]. This aspect was assessed using the Mini Mental Examination Test (MMSE) [49, 51–53, 58, 74, 76, 79, 81, 84, 85, 87–90, 92, 94–96, 109, 134], Global Deterioration Scale (GDS) [23, 47, 83, 85, 86, 135], Diagnostic and Statistical Manual of Mental Disorders (DSM III R) [48, 67], Dementia Severity Rating Scale (DSR) [80], Cambridge Mental Disorders of the Elderly Examination (CAMDEX) [48] and Clinical Dementia Rating Scale (CDR) [78]. The study samples represented a broad range of dementia severity, with five studies reporting mild dementia (MMSE ≤20) [51, 85, 90, 92, 134].

#### Theoretical framework

Approximately half of the studies (*n* = 43) reported a theoretical framework. The referenced theoretical models and frameworks are presented in Table 1. The original [22, 25, 136] and adapted versions of the BM were most often applied [27, 66, 69, 71, 73, 74, 78, 83]. However, approximately half of the included studies (*n* = 51) did not refer to a theoretical model [17, 46–48, 50, 51, 53, 54, 57, 59–61, 65, 68, 70, 75–77, 81, 82, 84, 86, 87, 90, 93, 94, 97, 98, 100, 103, 104, 106–110, 112, 115, 116, 120, 123–129, 131–134].

**Table 1:** Theoretical frameworks and models from sampled studies

Theoretical framework	Number of qualitative studies	Number of quantitative studies
Behavioural Model of Health Services Use (original and modified version) [22, 25, 136] *n* = 13		[23, 49, 52, 55, 56, 58, 63, 83, 85, 88, 89, 95, 96] *n* = 13
Framework for the study of access to medical care [154] *n* = 1		[69] *n* = 1
Expanded conceptual framework of the Behavioural Model [26] *n* = 3		[27, 66, 67] *n* = 3
Multiple sources concerning health service use *n* = 3		[71, 73, 78] *n* = 3
Theory of Reasoned Action [155, 156] *n* = 5	[111, 117–119] *n* = 4	[74] *n* = 1
Theory of Planned Behaviour in extension of the Behavioural Model *n* = 4	[117–119] *n* = 3	[74] *n* = 1
Model of caregiver stress [157] *n* = 2		[28, 91] *n* = 2
Theory of Health as Expanding Consciousness [158, 159] *n* = 2	[101, 102] *n* = 2	
Conflict-Theory Model of Decision-Making [160] *n* = 1	[122] *n* = 1	
Ecology model of adaptation and aging [161] *n* = 1		[62] *n* = 1
Behavioural Model and Practice-oriented conceptual framework for service use [162] *n* = 1		[63] *n* = 1
Self-developed conceptual model: cultural factors and respite use [64] *n* = 1		[64] *n* = 1
Help-seeking model [163, 164] *n* = 2	[113] *n* = 1	
Barrier concept/framework [165, 166] *n* = 2	[114] *n* = 1	
Sense of coherence [167] *n* = 1		[72] *n* = 1
Sociocultural Health Belief Model [168] *n* = 1	[121] *n* = 1	

#### Critical appraisal

The overall reporting quality of the studies was good to excellent. Details of the critical appraisal are presented in Table 2. A quarter of the studies (*n* = 24) fulfilled all quality criteria, nearly half (*n* = 43) achieved a score of 75% and approximately a quarter (*n* = 26) achieved a score of 50%. One mixed method study [132] achieved a score of 25%.

**Table 2:** Study quality according to MMAT

Study Type		Criteria		Number of Articles	
		Present	Absent	Not mentioned	Not applicable
Qualitative (*n* = 35)	1.1 Relevant source of data	35	0	0	0
	1.2 Relevant methods of analysis	33	0	2	0
	1.3 Context	31	3	1	0
	1.4 Reflexivity	14	18	3	0
Quantitative randomised (*n* = 1)	2.1 Randomisation	1	0	0	0
	2.2 Blinding	1	0	0	0
	2.3 Complete outcome data	0	1	0	0
	2.4 Dropout rate	0	1	0	0
Quantitative non-randomised (*n* = 16)	3.1 Selection bias minimised	15	1	0	0
	3.2 Appropriate measurements	15	0	1	0
	3.3 Comparable groups	11	0	1	4
	3.4 Complete outcome data/acceptable response rate	7	3	6	0
Quantitative descriptive (*n* = 38)	4.1 Sampling strategy	37	0	1	0
	4.2 Representativeness	24	9	5	0
	4.3 Appropriate measurements	38	0	0	0
	4.4 Acceptable response Rate	10	14	12	2
Mixed methods (*n* = 4)	5.1 Justification of design	4	0	0	0
	5.2 Data integration	1	0	2	1
	5.3 Limitations of integration	1	1	1	1

### Investigated formal care services

The included studies investigated different numbers of formal care services or mentioned no specific services (Table 3). Day care, home care and home help were the most frequently studied formal care services. Approximately a third of the studies investigated respite care, and a quarter investigated counselling services. Educational services were rarely investigated, and these studies targeted informal caregivers only. Several studies investigated a broad range of services, including support groups, transportation services, meals-on-wheels, or activating assistance.

**Table 3:** Investigated formal care services

Investigated services	References	Total numbers of references
One service	49, 50, 52, 54, 61, 63, 70, 85, 87, 91,92, 94, 95, 101, 104, 106, 108, 114, 117–119, 131, 133	23
Two services	23, 46, 51, 59, 64, 74, 90, 93, 98, 109, 129	12
Three services	55, 66, 71, 79, 111, 125	6
Four services	47, 48, 53, 57, 58, 76, 80, 102, 123, 128	10
Five or more services	17, 27, 28, 62, 67–69, 72, 73, 75, 77, 83, 84, 86, 88, 89, 96, 99, 127, 134,135	21
No specific type of service	56, 65, 78,81, 82, 97, 100, 103, 105, 107, 110, 112, 113, 115, 116, 120–122, 124, 126, 130, 132	22

### Investigation of the aspects influencing access to and use of formal community care

We analysed the main topics investigated by the included studies. Some studies investigated two main topics [52, 64, 74, 79, 113], and this was considered in our overview of the investigated influences (Table 4).

**Table 4:** Investigated influences

Main topic n = 94	Quantitative studies	Qualitative studies	Mixed method
Attitudes towards services *n* = 18	[17, 51, 71, 73, 74, 78, 87, 91] n = 8	[103, 106, 107, 111, 117–119, 128, 129] n = 9	[131] n = 1
Ethnicity *n* = 14	[28, 46, 52, 64, 88, 94] n = 6	[97, 108, 113, 121, 123, 125, 126, 130] *n* = 8	
Various influences *n* = 12	[48–50, 59–61, 65, 77, 82, 86, 92] *n* = 11		[134] n = 1
Influences according to the BM/adapted BM *n* = 10	[23, 27, 58, 63, 66, 67, 69, 74, 83, 89] n = 10		
Region of residence *n* = 7	[54, 55, 64, 80] n = 4	[99, 115, 116] n = 3	
Gender n = 7	[56, 79] n = 2	[100–102, 105, 124] n = 5	
Experiences with services *n* = 5		[82, 109, 110, 120, 122] n = 5	[132] n = 1
Early-onset dementia *n* = 5	[47, 57, 68] n = 3	[98, 126] n = 2	
Recommendations of healthcare professionals *n* = 4	[93, 95, 96] n = 3		[133] n = 1
Living alone *n* = 4	[52, 53, 84, 90] n = 4		
Barriers to service use *n* = 3	[62] *n* = 1	[104, 127] n = 2	
Needs of people with dementia or informal carers *n* = 3	[70, 81, 85] *n* = 3		
Financial factors *n* = 2	[75, 76] *n* = 2		
Religiousness *n* = 2	[79] *n* = 1	[113] n = 1	
Psychosocial factors *n* = 1	[72] *n* = 1		

### Attitudes towards services

Attitudes towards services comprised the most frequently investigated main topic in our sample (*n* = 18). Some studies focused on this topic by considering a specific type of service, such as respite care [74, 87, 106, 118, 119] or day care [51, 91, 111, 117, 131]. Other studies investigated the general attitudes of people with dementia and/or informal caregivers towards community services [17, 71, 73, 78, 103, 107, 128].

All except one study [51] investigated the attitudes of informal caregivers. Only the willingness to accept help was investigated in people with dementia [51], and this was found to increase as the duration of memory problems worsened. By contrast, studies on informal carers investigated a range of attitudes, from which three themes can be derived: attitudes towards services, inner barriers and cultural aspects.

Studies addressing attitudes towards services described positive or negative service beliefs [74, 117, 118]. Positive attitudes concerning service use were included in the psychological and social factors predictive of the use of increasingly diverse services [17]. In an example of positive service beliefs, informal carers agreed that service providers could provide equal- or higher-quality care, compared to that provided by family members [73]. Negative beliefs to use services included the caregivers’ feelings of guilt about placing their relatives with dementia in care [91, 119]. Other attitudes regarding services were related to aspects of the involved professionals. Proactive professionals were seen as crucial to encouraging informal caregivers to become actively engaged with formal support services [128]. By contrast, informal caregivers stated that their general practitioners (GPs) were not sufficiently aware of the available services [103]. Other studies investigated attitudes towards services related to the experiences of informal caregivers with service providers (e.g. inflexible services) that provided more burdens than support [122], or confusing service fragmentation [116]. In another study, participating informal caregivers [128] indicated that they would be willing to share their experiences and thus contribute to the education of service providers.

Several studies mentioned the inner barriers of informal carers, including the recognition of a problem as a prerequisite for help-seeking [100], or the utilisation of support services as a last resort when a crisis related to dementia placed a high burden on the informal caregiver [86]. Regarding the frequently described reluctance of people with dementia to accept professional support [51, 103, 107, 129], informal caregivers used several strategies to encourage their relatives with dementia to use special services (e.g., day care and/or short respite care) [129]. These strategies ranged from ‘allowing the person with dementia to decide whether to use the services’ to ‘coercing the person with dementia to use the services’.

The attitudes of informal carers may depend on their cultural backgrounds. For example, Asian-American informal caregivers are prevented from seeking help by an influential sense of shame [123] and Latino informal carers commonly misperceive even significant memory loss and disorientation as normal characteristics of age [108] or attach strong stigmas to AD [130].

Three studies used the Community Service Attitude Inventory (CSAI) by Collins et al. [137]. This standardised measurement of attitudes [71, 73, 87] comprises five components: concerns for the opinions of others, confidence in the service system, preference for informal care, belief in the informal caregiver’s independence and acceptance of government services. No significant differences in the attitudes of informal carers were observed between the users and non-users of respite services [87], and the lack of effect on the use of community services [73] suggested that although attitudes may play a particularly important role in explaining the use of discretionary services (e.g., domiciliary services), they are less important to an understanding of the use of nondiscretionary services (e.g., personal care). Families have considerable options in terms of the use of discretionary services, whereas the use of non-discretionary services is primarily dictated by the physical condition of the family member [136].

### Ethnicity

Sixteen studies addressed the effect of ethnicity on various groups of subjects in caregiving situations, including informal caregivers of Asian ethnicity in the USA [94, 104, 123, 130], people with dementia and informal caregivers of Asian ethnicity in the UK [125, 126] or Hong Kong [97] and African Americans [52]. Other studies investigated potential differences in caregiving and help-seeking between ethnic groups [28, 46, 64, 88, 112, 113, 121]. The influences of cultural attitudes and beliefs were described several times, as in the influence of the level of acculturation on attitudes towards service providers [121, 130]. Traditional values of respect towards older people [64, 113], concepts of normal aging [101, 108, 125] and family obligations to provide care [97] should be considered when developing services for people from minority ethnic groups. Additionally, certain ethnic groups may experience barriers related to their socioeconomic status [28, 94, 108, 123, 130]. A comparison of informal caregivers from four minority ethnic groups in the UK [112] found that socioeconomic status, rather than ethnicity, may better indicate the number of stress factors influencing access to services. Other studies described barriers related to language problems [108, 121, 123, 130] or waiting lists for services [88, 123].

### Various influences

Several studies investigated the access to and utilisation of formal community services without a main focus (*n* = 12). Five of these study reports were based on a German cross-sectional study [49, 50, 59–61]. Each study report addressed a specific type of community service: day care, short-term residential care, home nursing and home help, counselling and support groups for informal caregivers. All of the analyses identified the extent to which the service helped the individual situations of the informal caregivers as the main predictor of the utilisation of all these services. The sociodemographic factor ‘age of the person with dementia’ was found to be a predictor only of the use of home nursing and home help services. Some studies investigated a range of sociodemographic, health-related, support-related, psychological, or psychosocial influences [48, 65, 77, 82, 86, 92, 134]. Other studies describe the influence of specific factors on service utilisation. More severe dementia predicted a higher level of care [48, 77, 134]. Burden of caregiving was identified as a trigger for the use of formal care [65, 134]. Increasing problems when coping with ADL or loss of independence were positively associated with service use [48, 65, 77, 92]. Different results were found concerning the influence of depressive symptoms on the informal caregivers [77, 92]. Behavioural aspects were identified as strong predictors of service use [77, 86]. Somatic disorders or acute somatic events predicted higher levels of formal care [48, 65]. The relationship between the person with dementia and the informal caregiver influences the use of formal care. In particular, married men with dementia received less formal care [48, 92]. A lack of knowledge regarding the availability of services was identified as an important support-related influence [82, 86]. Therefore, informal caregivers wanted healthcare professionals to be better informed about dementia [82]. Vice versa, the informal carers’ lack of knowledge about the availability of services was one of the main reasons for low utilisation [86].

### Influences according to the BM/adapted BM

Ten studies used the BM to identify influences on the use of either a range of community services [23, 27, 58, 66, 67, 69, 83, 89] or only respite services [63, 74]. All 10 studies included predisposing, enabling and need factors. Although several predisposing factors were used, especially the sociodemographic characteristics of the participants, these factors were rarely described as important influences on service use. Some enabling factors were included, such as the availability of informal care, which seems important when combined with other influences. For example, informal care was found to strongly predict the non-use of respite care if the informal caregiver was a spouse living with the person with dementia [23]. One study found that enabling variables, such as the knowledge of and barriers to service use, availability of health insurance, informal support and others, could explain more variance in service use than either predisposing or need variables [83]. Additional enabling resources were confirmed as predictors of adult day care use [27]. Need factors were identified as important influences on service use, particularly the severity of behavioural problems [58, 62], need for ADL-related support [23, 58, 63] and the health and functional statuses of the person with dementia and the informal caregivers [69]. One study that included enabling and need factor variables of service use as predisposing factors identified an important influence of knowledge regarding the availability of services [67].

An adapted version of the BM was used to test interactions between informal caregivers’ enabling and need factors, and the need factors of the person with dementia [66]. For example, using day care might meet the need for relief for the informal caregiver. On the other hand, the informal caregiver might feel distressed by the need of the person with dementia to stay at home and might then experience discomfort if day care is utilized. Furthermore, an adapted version of the BM expanded the predisposing factors to include different types of caregiver beliefs (health, behavioural, control and normative beliefs) [74] derived from the Theory of Reasoned Action [138] and Theory of Planned Behaviour [29]. According to that study, the belief that service use would result in negative outcomes for the person with dementia was strongly associated with the non-use of day care and respite care, and was more strongly associated with service non-use than other predisposing, enabling and need variables.

### Gender

Seven studies investigated gender-related aspects, such as differences between male and female caregivers [79, 100] or the special situations of caregiving women [101] or men [102, 105, 124]. One study focused on differences in service use between men and women with dementia [56]. Three reports [100–102] were based on the same qualitative interviews of the elder spousal informal caregivers of older people with dementia. Among these caregivers, husbands began to seek help at an earlier point, compared to wives, and were more likely to view themselves as care managers than as informal caregivers [100]. These husbands tended to pass the responsibility for care and decision-making to others, usually a daughter or another woman. By contrast, wives tended to minimise the problems that they experienced and were likely to deny that these problems were too difficult to handle without help [101]. Regarding caregiving men, help-seeking was described as a process by which concessions were made for care, with steps to overcome both inner (e.g., personal values, beliefs, or characteristics) and systemic barriers [105]. For example, male informal caregivers may hold inner barriers to help-seeking such as the sense that no one else could do as good a job [124]. Gender differences in formal service use were found to depend on the type of service [79]. Specifically, male informal caregivers used more in-home services, whereas female informal caregivers used more transportation services; however, no gender differences were observed in the use of day care and support groups. Regarding people with dementia, women reported receiving more services and rated the availability of community services relatively higher, compared to men; however, women also reported greater unmet home care needs [56].

### Region of residence

Eight studies mainly addressed the region of residence. Although the majority of these studies addressed rural areas [54, 80, 99, 116, 132], others compared rural and urban areas [55, 64, 115]. One study discussed the term ‘rural areas’ from the viewpoint that such areas tend to be characterised by population dispersal over a large geographical area, which makes the provision of services more difficult [99]. However, an understanding of service provision in rural areas requires a more comprehensive view of the particular economic, social and political geographies of each location. Several barriers to support services in rural areas have been described, including transportation problems [115], restricted service options [54, 99, 116] and the roles of GPs who, despite the expectations of informal caregivers, did not always fulfil the role of the first gatekeeper [54] or a partner in dementia care management [116]. A comparison of cultural influences on the use of respite services by informal caregivers in rural and urban settings investigated geographic location as a cultural factor [64]. Notably, informal caregivers in urban areas had higher scores regarding respect for older people, which predicted an increased use of respite care; however, the authors could not explain this outcome. A study of the influence of stressors on help-seeking behaviours suggested that the type and extent of services used by rural families relied largely on needs related to the cognitive and functional abilities of the person with dementia [80]. Informal caregivers in rural areas either experienced required care as unavailable or lacked knowledge about accessing the services [55]. A study from North Wales found that all informal caregivers described informal help from neighbours as important [132]. The authors recommended acknowledging the roles of these informal helpers in normalizing support for caregivers to make this a culturally valued expectation.

### Experiences with services

Five studies explored the experiences of people with dementia and informal caregivers with services; these focused on the organisation of dementia care in Malta [110] and Canada [122], information pathways into dementia care services, service-related needs [109] and family experiences in the trajectory of the disease [82]. Four common themes emerged from these studies: timely support, comprehensive assessment, appropriate information and consistency of care providers. Timely support implies that informal caregivers expect to be involved in the assessment procedure and care planning [122], and would otherwise be ambivalent about the type of help they would need [110]. Other findings related to a delay in diagnosis suggest that health care professionals should more strongly emphasise the early recognition and validation of concerns over changes in the cognitive skills of individuals [109]. Informal caregivers expected a comprehensive and thorough assessment that would address not only the physical, but also the cognitive, psychological, social and spiritual needs of the person with dementia [122]. The assessment procedure should also include the needs of the informal caregivers. One study suggested well-defined care pathways to improve communication and coordination between service providers [109]. Others mentioned the limited knowledge about dementia and lack of awareness of available services [110, 120, 122]. A single point of access for providing information and coordinating services was suggested for the management of poorly coordinated available services and to facilitate more efficient and effective service delivery [109]. Although GPs were designated as the first point of contact and were potentially well placed to provide the necessary information and direction to care services, some informal caregivers described the information and direction they received from GPs as minimal to grossly inadequate [120]. In several studies, informal caregivers described their experiences with the inconsistency of care providers; for example, inadequately prepared care providers represented a main barrier to care [82]. Informal caregivers were unsure about the purposes of, eligibility for, or organisation of services [120]. Inconsistent care providers made it difficult for both the person with dementia and the informal caregiver to develop trusting, partnering relationships with the service providers [122].

### Early-onset dementia

Five studies addressed the situations of people with early-onset dementia or their informal caregivers [47, 57, 68, 98, 126]. The perspectives of the affected people were investigated qualitatively through individual interviews [98] and a case study [126]. The interviews included service-related questions such as: ‘Is/are there any help/services available for you?’ and ‘What are your opinions of these services?’ In the case study, the person with early-onset dementia described his perceptions of and experiences with help-seeking. In the individual interviews, participants spoke about the opportunities for socialisation and engagement in meaningful activities provided by day-care services. People with early-onset dementia experienced the risk management provided by professionals as a restriction of their wishes to remain independent for as long as possible. In the case study, the person with early-onset dementia considered trusting relationships with service providers to be important and was comfortable discussing his mental illness with professionals, but not with family members or friends. Two studies of early-onset dementia focused on the perspectives of informal caregivers [57, 68]. Here, the study participants thought that services did not meet the specific needs of people with early-onset dementia, which was identified as a critical aspect of service utilisation. One study used predefined influencing factors to determine that disease severity, behavioural problems and lower ADL scores led to more formal care utilisation [47]. More than half of the participants with early-onset dementia in this study were assessed as being mildly or moderately affected by dementia, according to the Global Deterioration Scale. The various methods applied in different studies revealed that early-onset dementia led to noticeable differences in the understanding of influences on service utilisation.

### Living alone

Four studies analysed the influence of living alone by comparing people with dementia who lived alone to those with a co-resident (usually a family member). One study each evaluated a case management programme [52] and care management programme [53]. A third study compared the long-term cognitive and functional abilities of solitary people with AD relative to those living with a co-resident [90]. A further study compared service use patterns in a sample of people with dementia who lived alone versus those who lived with a spouse [84]. People with dementia who lived alone tended to be older than their counterparts with co-residents, and were predominantly female [84, 90]. However, people who lived alone and with a co-resident had very similar cognitive statuses [84, 90]. For people with dementia who lived alone, moderate-to-severe dementia was identified as a significant predictor of receiving less formal support [52]. System-related influences were strongly associated with access to support services. For example, the current service delivery system in the USA was designed to meet the needs of older adults with family support, rather than those living alone. By contrast, people in Sweden received formal care according to their actual needs and independently of their household status, given the publicly funded nature of services in that country [90]. In other national contexts, living alone with dementia is possible; however, service providers should consider these research findings when planning future developments [53].

### Recommendations by healthcare professionals

Three studies highlighted recommendations by health care professionals [93, 95, 96]. One study from Germany investigated the influence of recommendations by trained GPs on the utilisation of support services [93]. A 2-year follow-up evaluation revealed an increased utilisation only of support groups, whereas no other services had been accessed. Several reasons for the low utilisation rate were discussed, including the finding that two-thirds of participants had mild dementia at the beginning of the study, and the inappropriateness of the available support services was indicated. Two studies investigated recommendations by the California Alzheimer’s Disease Diagnosis and Treatment Center (ADDTC) on the use of adult day care [95] and community care [96]. Both studies showed only minor effects of this recommendation on utilisation rates. One study implied that policy makers and program administrators should reassess aspects of the ADDTC program, which did not emphasise recommendations for support services [95]. These recommendations might have been inappropriate or may not have accommodated the needs of people with dementia and their families [96]. Otherwise, individual-level reasons, such as the acceptability of recommendations by informal caregivers [95] or financial barriers, were presumed [96]. These three studies similarly observed a weak effect of recommendations on utilisation rates, for which various reasons were given. In a study concerning the role of secondary support in mediating formal services for dementia, informal caregivers described mediation as a special type of recommendation in which information, encouragement and instrumental support activities are provided [133]. In that study, the presence of a formal or informal mediator was predictive of contact with respite services, but not other types of services.

### Needs of people with dementia or informal caregivers

Three studies focused on need aspects. One study used the Camberwell Assessment of Need for the Elderly (CANE) [139] to investigate the needs of people with dementia and the connection to service use [85]. The CANE instrument assesses met and unmet needs in social, medical, psychological and environmental areas. Between people with dementia and informal caregivers, the CANE yielded a poor-to-moderate degree of agreement regarding needs and a fairly good agreement regarding the actual extent of professional help. In terms of unmet needs for memory, daytime activities and psychological distress, both people with dementia and informal caregivers agreed that they were unaware of the availability of relevant services. Other reasons for unmet needs included refusal by people with dementia, excessive bureaucracy, unclear information and others. One qualitative study emphasised service-related needs [109] by interviewing both people with dementia and their informal caregivers. The identified crucial service-related needs included early diagnosis, access to well-coordinated care, continuity of involved personnel and access to non-pharmacological interventions to support identity and social engagement. Two studies addressed the influence of the burdens of informal caregivers on the utilisation of services [70, 81]. Informal caregivers who provided care to people with dementia-related behavioural disturbances were significantly more likely to develop a high caregiving burden level, compared to other informal caregivers [81]. However, the burden level was not identified as a factor influencing service utilisation. Another study investigated the use of day-care services by highly burdened informal caregivers [70]. Here, the availability of informal help was the only variable predictive of the use of day-care services, whereas sociodemographic variables had no effect.

### Barriers to services use

Three studies focused on the barriers to [104] or criticism of support services [127], as well as factors associated with the non-use of services [62]. The informal caregivers mentioned barriers such as the failure of GPs to provide sufficiently early referrals for services [104], which may be attributable to physicians’ lack of knowledge regarding the services or expertise in dementia care [127]. Differences in the perceptions of useful services between informal caregivers and service providers were described as reasons for the non-use of services. Similarly, informal caregivers described feeling that they were misunderstood by health care professionals in terms of the experienced burden level [104]. Informal caregivers had little knowledge of available services [62] and they experienced difficulties in obtaining information about services because of the complicated service system [127]. Additionally, the competence of the informal caregivers in terms of providing care was a significant barrier to service use [62]. This means the informal carers’ estimation of their personal caregiving competence influenced their decisions not to use services. Therefore, health care professionals should place the highest priority on the development of caregiving competencies.

### Financial aspects

Two studies investigated financial influences related to support service utilisation. A French study [76] provided evidence supporting an association of public financial support with greater total care expenditures. The proportion of formal care relative to informal care use was greater among people with dementia who received financial support. Furthermore, a Scottish study compared the financial burdens of providing care to people with and without dementia [75]. Informal caregivers of people with dementia were more engaged in caregiving than were their counterparts, and household expenses were higher in the dementia caregiving group, which used more domiciliary and day-care services. Although other studies did not focus mainly on financial factors, several mentioned the influence of the involved costs as a barrier to formal support [9, 28, 55–57, 59, 66, 102, 106, 108, 110, 122, 131].

### Religiousness

Two studies focused on religiousness and its interactions with other aspects, such as the influences of gender and religiousness [79] and the role of religion and ethnicity [113]. In general, women received higher scores on measurements of religiousness, which indicated greater involvement in religious activities, more frequent prayer or meditation and increased valuation of spirituality. However, the influence of religiousness on service use was observed only for transportation services, but not for day-care or support services [79]. An investigation of four different ethnic minority populations in the USA (African-American, Chinese-American, Irish-American and Puerto Rican) found that ethics related to care provision were associated with the religious attitudes of the informal caregivers [113]. For example, some Catholic informal caregivers considered caregiving to be a duty, and used prayer to deal with the associated burdens. Participants in all informal caregiver groups except Chinese-Americans reported these attitudes.

### Psychosocial aspects

Only one study investigated psychosocial factors using the concept of ‘sense of coherence’ (SOC) and the level of stress related to the behavioural disturbance of the person with dementia [72]. The SOC describes the utilisation of psychological, social and cultural resources based on several coping strategies intended to resist ill health. A comparison of the users and non-users of mental health services showed that informal caregivers in the latter group scored significantly higher on the SOC measurement scale, indicating a higher competence regarding stressful situations [72].

### Relation of the identified influencing factors to the BM

We then focused on the identified main topics of the studies and determined whether these corresponded with the factors and domains of the adapted BM. Most main topics could be allocated within the psychosocial, enabling and need factor categories (Table 5). However, other main topics did not fit into the model, including sociodemographic factors such as gender, ethnicity, living alone, religiousness and region of residence. These sociodemographic factors corresponded with the predisposing factors included in the original BM.

**Table 5:** Relation with the BM

Factor	Domain	Main topic of the studies
Psychosocial	Attitudes	- Attitudes towards services - Experiences with services
	Knowledge	
	Social norms	
	Perceived control	- Psychosocial factors (Sense of Coherence)
Enabling	Availability of support	- Recommendations of health care professionals - Barriers to service use - Multiple predictors: support related influences
	Financial resources	- Financial factors
Need	Objective	
	Perceived	- Needs of people with dementia and informal carers - Early-onset dementia - Multiple predictors: severity of dementia, ADL problems, depression, behaviour, comorbidities, perceived health status, burden

The adapted BM mainly focuses on the domain ‘Psychosocial influences on service use’, which was described in more than two-thirds of the included studies. We could categorise most described psychosocial influences according to the domains in the adapted BM [30]: ‘Attitudes’, ‘Knowledge’, ‘Social norms’ and ‘Perceived control’. Manifold themes emerged within the psychosocial influencing factors and provided an impression of the range and complexity of these influences.

Attitudes as the main theme of several included studies were described above.

The studies frequently mentioned knowledge-related influences, which might be sub-categorised as a lack of knowledge about dementia and services, the knowledge of health care professionals and knowledge-related barriers. A lack of knowledge about dementia was described as common among South Asian communities in the UK [125], and Belgian informal caregivers confirmed a need for more knowledge about dementia [17]. In a Dutch study, people with dementia and informal caregivers stated that they were unaware of relevant services [85]. Several studies also mentioned a lack of knowledge about available services [66, 67, 75, 91, 107, 122]. In contrast, a study conducted in the USA [54] found that only a few informal carers lacked knowledge about available services. Many publications have described aspects related to the knowledge held by professionals. One study described recommendations by GPs intended to motivate informal caregivers to use support services as a successful strategy [93]. However, other studies found that health care professionals provided inadequate information [102] or that staff members were poorly informed about dementia and dementia care [82]. Occasionally, the information or proposals for care or treatment suggested by health care professionals were disregarded by people with dementia or informal caregivers [134]. In addition to making services available, professionals should also inform people with dementia and informal caregivers about these services [63]. Several difficulties related to knowledge aspects were described. For example, informal caregivers reported that they did not know what type of advice was needed [110] or were unable to obtain information at the right time [120].

The findings related to the domain ‘Social norms’ were categorised as family and cultural obligations and normative beliefs. The influence of family obligations on caregiving roles has been described several times, and some studies investigated a cultural basis for family obligations. One such study investigated the help-seeking behaviours of Chinese informal caregivers, and reported the unwillingness of these individuals to disclose family problems to people outside of the family [97]. Two additional studies mentioned the influence of family obligations on informal caregivers with an Asian background [67, 123]. These influences should also be considered in light of gendered expectations [110] and generational differences [130]. Canadian informal caregivers described a sense of obligation and commitment to fulfilling the caregiving role as their ‘role in life’ [122]. Normative beliefs were found to act as barriers to service use [67]. Informal caregivers felt they were ‘not coping’ with their caregiving responsibilities if they sent their relative with dementia to a day-care centre [117]. Other informal caregivers described such normative beliefs as a sense of duty towards their partners with dementia [103].

Aspects of the domain ‘Perceived control’ were related to the topics of reluctance to use services, independence and decision-making. Several studies investigated the reluctance of people with dementia to use services [51, 62, 74, 85, 103, 118, 123, 129, 132, 135]. People with dementia were reluctant to use day-care services because they believed that they did not need this supervision, they liked being on their own and expected that they would not enjoy it [51]. Informal caregivers were also found to refuse support services [103, 106, 115, 135], for reasons ranging from pride to the wish to manage care independently to feelings of guilt for accepting professional support [103]. The maintenance of independence was important to both people with dementia and informal caregivers. Younger people with dementia explained their fears about professional risk management, which might inadvertently remove their independence at an earlier stage than necessary. Male informal caregivers reported maintaining independence as among the most important reasons why they would not seek help [105]. This reason also appears to be important for informal caregivers with traditional ideologies, which include an attempt to control the caregiving situation [112]. One study used an independence-related scale, the Belief in Caregiver Independence subscale of the CSAI, to determine that these and other measured beliefs were related significantly to caregiving experiences [78]. The decision-making aspects of service utilisation were described in different ways. For example, informal caregivers demanded to be included as equal partners at the intersection of formal and informal care systems [128]. Informal caregivers from several cultural backgrounds described different decision-making processes in their families, thus indicating that service providers should recognise heterogeneity within different ethnic groups [113]. A BM-based investigation of service use concluded the requirement for a more sophisticated model of decision-making processes, in which informal carers determine when services are needed [58]. Both people with dementia and informal carers often experienced difficulty with decision-making, which originated from their lack of awareness of treatment and care options [134].

Within the adapted version of the BM, enabling factors include the categories ‘Availability of support’ and ‘Financial aspects’. The domain of ‘Availability of support’ comprises the identified main topics of recommendations of health care professionals, barriers and other support-related influences. The enabling domain ‘Financial aspects’ was the main topic of two studies and a sub-theme in several additional investigations. In the adapted BM, need factors include the categories ‘Objective needs’ and ‘Perceived needs’, as in the original BM. The included studies did not identify the category of ‘Objective needs’, a consequence of the exclusion criteria applied to this review. ‘Objective needs’ have been described as clinical evaluations. Therefore, we excluded studies that investigated the attitudes, meanings and evaluations of health care professionals regarding the access to and utilisation of formal community care for dementia. In contrast, ‘Perceived needs’ were described as the subjective estimations of users or non-users of health care services. Some included studies focused on related topics such as the needs of people with dementia and informal caregivers, needs related to early-onset dementia, and other special needs related to dementia severity, ADL problems, depression, behaviour, comorbidities, perceived health status and informal caregiver burdens.

## Discussion

This review included 94 studies of the factors influencing the access to and utilisation of formal community care for dementia. We identified a wide range of aspects influencing the use of professional support by people with dementia and their informal caregivers and families. A critical appraisal of the included studies using the MMAT indicated that two thirds of the studies received scores of at least 75%.

We investigated a range of community care services, including day care, respite care, home care, home help, counselling and others. The first four services were investigated more frequently than were other services such as education, counselling, or support groups. These differences in prioritisation of dementia-related health service research may be attributable to several factors. The services are likely to solicit different levels of acceptance. For example, services that support people with dementia and physical disabilities seem to be more acceptable to both people with dementia and informal caregivers [6]. Day care, home care, home help and respite care services tend to address physical needs relatively better than services such as education, counselling or support groups. By contrast, informal caregivers described challenges in learning how to seek non-medical support [128], or reported their inability to access appropriate services and being actively turned away by service providers [140]. In a Dutch study, both people with dementia and informal caregivers described met needs related to food and household activities, but unmet needs related to memory, information, company, psychological distress and daytime activities [85]. Consequently, it seems necessary to inquire not only about the reasons related to the access and utilisation of support services. Services should be available that are appropriate for the different needs. Furthermore, people with dementia and their informal caregivers should be motivated to use formal care and in accessing services, e.g. through early and constant contact to a health- or social-care professional [141].

The majority (*n* = 49) of the included studies investigated multiple types of service. However, only a few reported their findings related to service-specific criteria, such as in-home or out-of-home services or health and human care services [83, 135]. Study results refer to the importance of separating services or categories of services, because the predictors are different [142]. Few studies (*n* = 5) concerning support services for people with early-onset dementia were identified, and only two focused on service use from the perspective of this population. Even fewer studies investigated counselling services exclusively for people with dementia, and no identified studies focused on educational services for this population. We identified only one study concerning rare dementia types [126]. This case study followed the situation of a male person with a diagnosis of fronto-temporal dementia. He experienced difficulties in accessing formal care services, i.e. problems of transportation and accompaniment to and at support groups.

It is difficult to make a distinction between the different types of dementia since the greater part of research did not separately investigate one type of dementia (e.g. [143]).

These findings demonstrate that research on the influences of access to and utilisation of support services predominantly targets the informal caregivers and their perspectives. This contradicts Murphy [144], who explained that opinions had changed and informal caregivers were no longer considered the main users; rather, people with dementia were well placed to comment on the services they had received. The findings of a recent review indicate a lack of research representing the perspectives of people with early-onset dementia [145]. Instead, the literature suggests that people with dementia can express their needs, and that their perspectives should be included in research and service improvements [85]. However, when involving people with dementia in service development and evaluations, one must realise that service users will have different experiences with different services [146]. A recent review of proxy decision-making by the informal caregivers of people with dementia [147] found that the caregivers feel a responsibility to honour the previous wishes of the person with dementia. People with dementia often resist the use of services, which could delay formal care until a crisis occurs. Consequently, people with dementia have been excluded from decision-making. Evaluations of community care services have led to suggestions of caution regarding the differing perspectives and priorities of people with dementia and their informal caregivers if only the latter are relied on as proxy respondents [148, 149]. Dementia care providers must also proactively consider the perspectives of informal caregivers both during policy development and everyday practice to reduce the burden of care [128]. The findings from a Canadian study of the availability and accessibility of community care services suggest an integrated, continuing care model that includes both people with dementia and informal carers as partners in care [122]. A recent focus group study aimed to enhance the understanding regarding facilitators, barriers and the use of formal care services [150]; this means the perspective of people with dementia was included.

The traditional BM [22, 25] offers a framework for categorising investigated influences according to the main categories of predisposing, enabling and need factors. Examples of predisposing factors include attitudes towards services [e.g. 87, 103], living alone [52, 53], or the region of residence [54, 99]. Enabling factors included financial aspects [75, 76] or the influence of recommendations [93, 96]. Need factors were also investigated [70, 81, 85], e.g. the use of services increased as dementia progressed and physical disability worsened [81]. This range of influences seems to confirm previous research, which found that predisposing, enabling and need factors each influence the use of services among people with cognitive impairment; in other words, need factors are not predominant [151]. However, the need factors of burdens and stresses experienced by informal caregivers and the behavioural problems of people with dementia are frequently investigated [47, 97, 104].

We aimed to determine whether the investigated influences on service use could be categorised using the adapted BM, from which predisposing factors, especially sociodemographic characteristics, have been removed [30]. Therefore, the adapted form includes psychosocial aspects, which comprise attitudes, knowledge factors, social norms and perceived control beliefs. Although we identified several studies that focused on the influences of sociodemographic factors on service use, these aspects did not encompass the categories of the adapted BM. Studies that examined aspects such as ethnicity, living situation, region of residence, or gender reported noteworthy influences on service use. Therefore, it seems inappropriate to use a theoretical model that excludes these aspects. However, the data analysis step in which psychosocial aspects were extracted according to the adapted BM highlighted a variety of investigated influences that would likely have been overlooked when using the traditional BM. Although health beliefs are common predisposing factors in the traditional BM, the adapted version acts as a lens to magnify the details of psychosocial influences on service use. For example, the aspect of independence was mentioned several times as having an important influence on service use [78, 98, 105, 112]. The concept of formal care as a threat to individual independence was also emphasised in a qualitative section of the recent Actifcare study [150].

The ability of the adapted BM to show correlations between the categories seems to be limited. For example, support service use was initiated after a person with dementia became acutely ill, and the physician requested a publically financed nursing service [106]. This positive experience might encourage the patient to seek similar support in the future. The influencing aspects mentioned herein can be categorised as factors related to the availability of services, financial resources, attitudes towards services and objective needs. The above example demonstrates the difficulties of linking correlations between influences on service access and utilisation, which should be considered when applying the several versions of the BM. Regarding practice implications, different aspects should be regarded in context, and multiple aspects should be considered. For example, financial support programs require appropriate information and referral of the target groups. To further develop the BM, we suggest the integration of these two versions to offer an opportunity for considering the sociodemographic, psychosocial, enabling and need predictors of health and social care utilisation.

As an alternative to the BM, one could use the theoretical construct of help-seeking, which has been described as an active and adaptive process of attempting to cope with problems or symptoms by using external resources for assistance [152]. A review of help-seeking behaviours related to dementia found that most studies did not rely on theoretical frameworks; nonetheless, the psychosocial influences of inadequate knowledge and stigmatic beliefs were identified as the most important barriers to help-seeking [20]. To overcome these barriers, we suggest the more active involvement of people with dementia and informal caregivers in research related to help-seeking and service use, the further development of support services and daily decision-making related to living with the condition of dementia.

### Strengths and limitations

The present scoping review used a version of the frequently applied BM that was adapted for long-term care. Unfortunately, we found no publications for the application of the adapted BM in studies of caregiving in dementia. The model helped us to describe the identified psychosocial influences on the access to and utilisation of formal community care in dementia in more detail than the original BM. However, the adapted BM requires further development towards better consideration of the complex influences on service use.

Since there are no reporting guidelines dealing with the conduction of scoping reviews [153], it remains unclear how to best organise the broad spectrum of literature. We therefore focused on the main topics investigated in the literature, taking the risk of disregarding other important aspects. We critically appraised the studies in order to get insight into the trustworthiness of the research. We applied a critical appraisal tool to demonstrate not only the scope of the research, but also an impression of its validity. The MMAT could be applied the majority of included studies.

Our scoping review aimed to present a broad overview of the investigated influences on access to and use of formal community care in dementia. Since we included a large number of studies (*n* = 94) coming from 18 countries, it was not feasible to take into account each of the national healthcare systems where the research took place. This is certainly a limitation.

## Conclusion

According to the literature, psychosocial factors related to service use for dementia have been frequently investigated, and should be considered to understand the reasons for the use or non-use of professional help in relation to other influences at both the individual and systemic levels. More attention should be given to the active involvement of the perspective of people with dementia in research and the further development of services. This inclusion would not contribute to an understanding of the access to and utilisation of formal community care, but also to other important aspects of care and treatment for people with dementia, such as diagnostic procedures, treatment options and aspects of the quality of life. We suggest further developing the theoretical background of health service use by people with long-term conditions. Therefore, all involved perspectives should be considered; in other words, the perspectives of health care professionals should be considered in addition to those of the people with dementia and informal caregivers. Further research might involve an overview of investigations regarding the perspectives and experiences of health care professionals on service use in dementia cases. Furthermore, political and societal efforts should consider the voices of people with dementia to ensure that appropriate support is available at the right time and in the right place.

## Appendix

**Table 6:** Description of factors of the expanded Andersen Model by Bradley et al. (2002)

**Factor**	**Domain**	**Description**
Psychosocial	Attitudes	An attitude toward a behaviour is the degree to which performance of the behaviour is positively or negatively valued. It is determined by the total set of accessible behavioural beliefs linking the behaviour to various outcomes and other attributes. Behavioural beliefs link the behaviour of interest to the expected outcomes and represent the subjective probability that the behaviour will produce a given outcome^a^.
	Knowledge	Instead of ensuring that people have accurate information, we should determine what information they actually possess and how this information affects their intentions and actions, irrespective of the accuracy of the information. Furthermore, we should be concerned about the information or knowledge guiding the behaviour of interest (i.e., beliefs about the behaviour), rather than about general knowledge in a behavioural domain ^b^.
	Social norms	Normative beliefs refer to the perceived behavioural expectations of important referent individuals or groups such as the person’s spouse, family, friends, teachers, doctor, supervisor and colleagues. It is assumed that these normative beliefs, together with the person’s motivation to comply with the different referents, determine the prevailing subjective norm. The subjective norm is the perceived social pressure to engage in or avoid a behaviour. The subjective norm is assumed to be determined by the total set of accessible normative beliefs related to the expectations of important referents ^c^.
	Perceived control	Perceived behavioural control refers to a person’s perception of their ability to perform a given behaviour. Perceived behavioural control is assumed to be determined by the total set of accessible control beliefs (i.e., beliefs about the presence of factors that may facilitate or impede performance of the behaviour) ^d^.
Enabling	Availability of support	Enabling conditions make health service resources available to the individual. These conditions can be measured according to family resources such as income, level of health insurance coverage, or other source of third-party payment, whether or not the individual has a regular source of care or the nature and accessibility of that regular source of care. Apart from family attributes, certain enabling characteristics of the community in which the family lives, such as the amount of health facilities and personnel in the community, can also affect the use of services. Other measures of community resources include the region of the country and rural/urban nature of the community in which the family lives ^e^.
	Financial resources	
Need	Objective	In addition to perception of illness by the individual or his family, the model also includes a clinical evaluation because once an individual seeks care from a formal system, the nature and extent of that care are partly self-determined ^e^.
	Perceived	Assuming the presence of predisposing and enabling conditions, the individual or his family must perceive the illness or the probability of its occurrence to use health services. The illness level represents the most immediate cause of health service use ^e^.

^a^Ajzen, I. (2006). TPB Diagram. Retrieved from: people.umass.edu/aizen/tpb.diag.html

^b^Ajzen, K., Joyce, N., Sheikh, S. & Cote, N.G. (2011). Knowledge and the Prediction of Behaviour: The Role of Information Accuracy in the Theory of Planned Behaviour. Basis and Applied Social Psychology, 33, 101–117

^c^Ajzen, I. (2006). TPB Diagram. Retrieved from: people.umass.edu/aizen/tpb.diag.html. The domain of social norms was derived from the construct of Normative beliefs and Subjective norms by the TPB Model

^d^Ajzen, I. (2006). TPB Diagram. Retrieved from: people.umass.edu/aizen/tpb.diag.html

^e^Andersen & Newman (2005). Societal and Individual Determinants of Medical Care Utilization in the United States. Health and Society, 51(1), 95–124

## Abbreviations

AD: Alzheimer’s Disease
ADL: Activity of daily living
BM: Behavioural Model of Health Service Use
CANE: Camberwell Assessment of Need for the Elderly
CSAI: Community Service Attitude Inventory
GP: General Practitioner
